# Investigation of Proposed Ladderane Biosynthetic Genes from Anammox Bacteria by Heterologous Expression in *E*. *coli*

**DOI:** 10.1371/journal.pone.0151087

**Published:** 2016-03-14

**Authors:** Pouya Javidpour, Samuel Deutsch, Vivek K. Mutalik, Nathan J. Hillson, Christopher J. Petzold, Jay D. Keasling, Harry R. Beller

**Affiliations:** 1 Joint BioEnergy Institute, 5885 Hollis Avenue, Emeryville, CA, United States of America; 2 Biological Systems and Engineering, Lawrence Berkeley National Laboratory (LBNL), Berkeley, CA, United States of America; 3 Joint Genome Institute, 2800 Mitchell Drive, Walnut Creek, CA, United States of America; 4 Environmental Genomics and Systems Biology, LBNL, Berkeley, CA, United States of America; 5 Department of Chemical & Biomolecular Engineering, University of California, Berkeley, CA, United States of America; 6 Department of Bioengineering, University of California, Berkeley, CA, United States of America; 7 Earth and Environmental Sciences, LBNL, Berkeley, CA, United States of America; Imperial College London, UNITED KINGDOM

## Abstract

Ladderanes are hydrocarbon chains with three or five linearly concatenated cyclobutane rings that are uniquely produced as membrane lipid components by anammox (anaerobic ammonia-oxidizing) bacteria. By virtue of their angle and torsional strain, ladderanes are unusually energetic compounds, and if produced biochemically by engineered microbes, could serve as renewable, high-energy-density jet fuel components. The biochemistry and genetics underlying the ladderane biosynthetic pathway are unknown, however, previous studies have identified a pool of 34 candidate genes from the anammox bacterium, *Kuenenia stuttgartiensis*, some or all of which may be involved with ladderane fatty acid biosynthesis. The goal of the present study was to establish a systematic means of testing the candidate genes from *K*. *stuttgartiensis* for involvement in ladderane biosynthesis through heterologous expression in *E*. *coli* under anaerobic conditions. This study describes an efficient means of assembly of synthesized, codon-optimized candidate ladderane biosynthesis genes in synthetic operons that allows for changes to regulatory element sequences, as well as modular assembly of multiple operons for simultaneous heterologous expression in *E*. *coli* (or potentially other microbial hosts). We also describe *in vivo* functional tests of putative anammox homologs of the phytoene desaturase CrtI, which plays an important role in the hypothesized ladderane pathway, and a method for soluble purification of one of these enzymes. This study is, to our knowledge, the first experimental effort focusing on the role of specific anammox genes in the production of ladderanes, and lays the foundation for future efforts toward determination of the ladderane biosynthetic pathway. Our substantial, but far from comprehensive, efforts at elucidating the ladderane biosynthetic pathway were not successful. We invite the scientific community to take advantage of the considerable synthetic biology resources and experimental results developed in this study to elucidate the biosynthetic pathway that produces unique and intriguing ladderane lipids.

## Introduction

**Fig 1:**
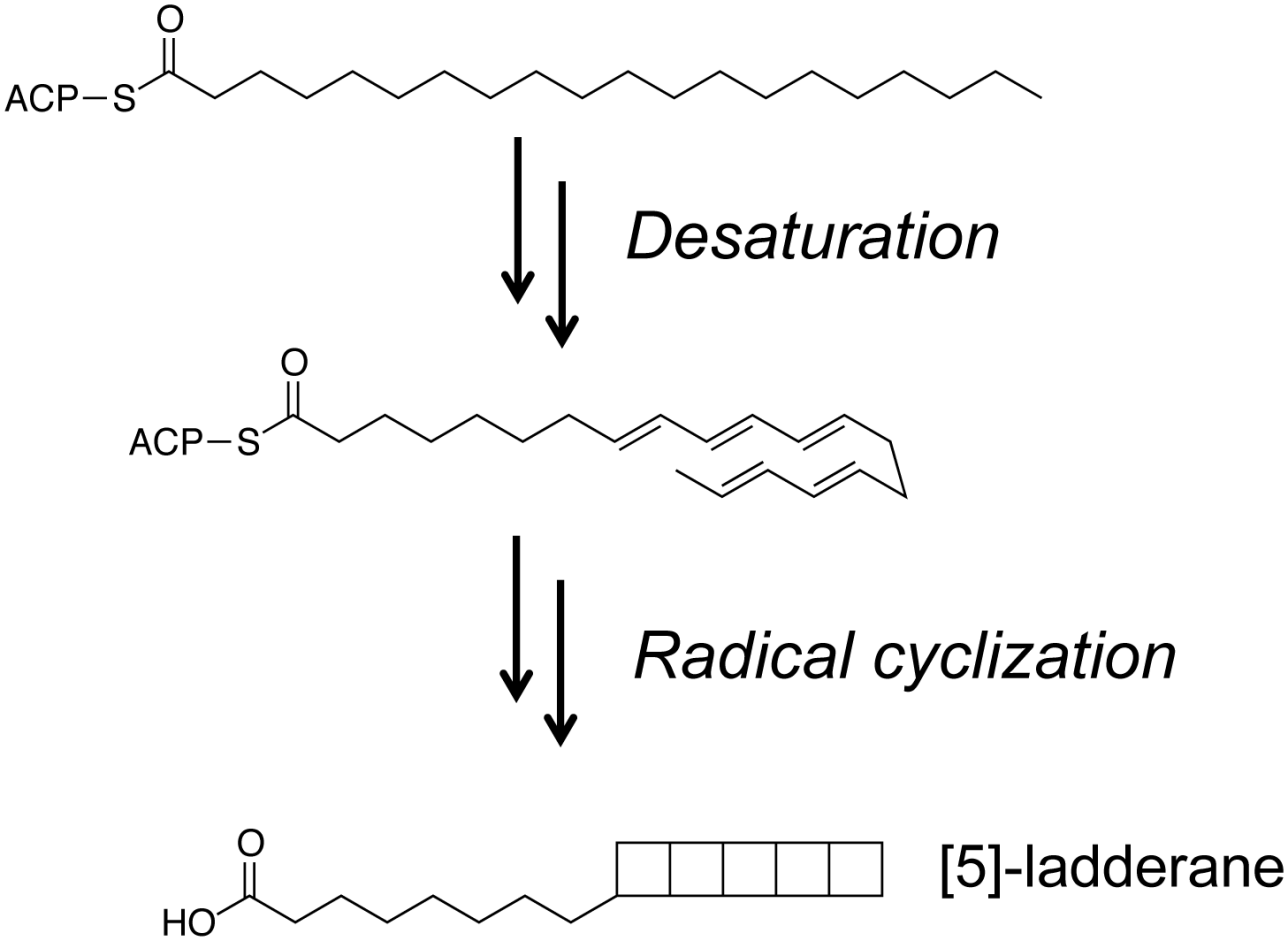
Structure of C_20_ [5]-ladderane fatty acid, and the proposed major steps of the ladderane biosynthetic pathway. desaturation of acyl-ACPs to form polyunsaturated (all-*trans*) intermediates and cyclization *via* a radical cascade mechanism (adapted from [11]).

Ladderanes (e.g., Fig 1) are hydrocarbon chains with three or five fused cyclobutane rings that are uniquely produced as membrane lipid components by anammox (anaerobic ammonia-oxidizing) bacteria [1–3]. Ladderanes are unusually energetic compounds by virtue of their angle and torsional strain [4, 5]. While renewable, microbially produced fuels derived from conventional fatty acids, such as fatty acid ethyl esters or medium-chain methyl ketones, have recently been developed and have favorable properties as diesel fuel blending agents [6–10], it is plausible that fuels derived from ladderane fatty acids would have excellent jet fuel properties, in particular, high volumetric energy density. For example, estimations of volumetric energy densities of ladderane structures suggest that a [5]-ladderane could have ~46% greater volumetric energy density than conventional jet fuel used in the U.S. (S1 Table), which would lead to greater energy efficiency.

The primary challenge in producing ladderane-derived fuels in a renewable fashion, for example, using engineered microbes to make them from cellulosic sugars, is that the underlying biochemistry and genetics of ladderane biosynthesis are unknown. Ladderane synthesis has been an enigmatic topic of fascination to synthetic organic chemists as well as biochemists [5, 11–13]. Although an elegant chemical synthesis of [5]-ladderane fatty acid (pentacycloanammoxic acid) was accomplished [12], the synthesis was laborious, low yielding (~2%), and required unconventional chemical feedstocks, making it poorly suited to scale-up. Biosynthesis of linearly concatenated cyclobutane rings has no precedent among known enzymatic reactions. However, Rattray and co-workers [11] have identified a pool of 34 gene candidates, some or all of which may be involved with ladderane biosynthesis. These 34 genes (occurring in four gene clusters) were identified in the reconstructed genome of the anammox bacterium *Kuenenia stuttgartiensis*, which dominated an enrichment culture whose metagenome was sequenced [13]; note that anammox bacteria have not yet been isolated in pure culture. The selection of the 34 candidate genes [11] was based in part on key hypothesized reactions in ladderane fatty acid biosynthesis, namely desaturation of an acyl-ACP to form a polyunsaturated (all-*trans*) intermediate and radical-mediated polycyclization of that intermediate to form the ladderane structure (Fig 1). Arguments that this group of genes could be involved in ladderane biosynthesis included the following: (i) the four gene clusters include canonical and non-canonical versions of fatty acid biosynthetic genes, suggesting their involvement in synthesis of fatty acid derivatives (such as ladderane fatty acids); (ii) the clusters include homologs of key putative gene products, namely phytoene desaturases and *S*-adenosylmethionine (SAM) radical enzymes, which could be involved in desaturation of acyl-ACPs and radical cascade cyclization, respectively; (iii) computational chemistry studies have indicated that ladderane cyclization could be mediated by radical chemistry, although polycyclization of a hydrocarbon with the unsaturation pattern shown in Fig 1 (middle) would be endothermic, and cationic polycyclization has also been proposed (by analogy to terpene cyclization) [5, 14]; and (iv) searches for polyketide synthases (PKSs), which could potentially catalyze synthesis of all-*trans* polyunsaturated compounds, in the *K*. *stuttgartiensis* genome yielded no positive results.

The purpose of the present study was to establish a means of testing the 34 candidate genes from *K*. *stuttgartiensis* for involvement in ladderane bioysnthesis through synthesis of the codon-optimized genes and heterologous expression in *Escherichia coli* under anaerobic conditions. *E*. *coli* was chosen as a host because it is a facultative anaerobe that can be grown fermentatively and because, to date, no anammox bacterial species have been isolated in pure culture and there is no genetic system established for any anammox bacterium [2, 15]. Furthermore, anammox bacteria are obligate anaerobes that have a generation time of approximately two weeks and require nonstandard cultivation conditions for growth [15], making these organisms intractable for biochemical studies of the proposed scope and magnitude. This study describes the design and assembly of synthetic operons including candidate ladderane pathway genes, as well as a method for soluble purification of one of the crucial enzymes in the hypothesized pathway, a desaturase. This study is, to our knowledge, the first experimental effort focusing on the role of specific anammox genes in the production of ladderanes, and lays the foundation for future efforts toward determination of the ladderane biosynthetic pathway.

## Materials and Methods

### Bacterial strains, plasmids, and reagents

Bacterial strains and plasmids used in this study are listed in Table 1. Strains and plasmids along with their associated information (annotated GenBank-format sequence files) have been deposited in the public instance of the JBEI Registry [16] (https://public-registry.jbei.org/folders/222; JBEI strain and plasmid numbers along with JPUB part IDs are given in Table 1) and are physically available from the authors and/or Addgene (http://www.addgene.org) upon request. Phusion DNA polymerase, restriction enzymes, and T4 DNA ligase were purchased from Thermo Scientific (Waltman, MA). Plasmid extractions were carried out using Qiagen Miniprep Kits (Valencia, CA). All organic solvents were purchased from Sigma-Aldrich (St. Louis, MO) and were pesticide-residue-analysis grade.

**Table 1:** Bacterial strains and plasmids used in this study.

Strain or plasmid	JPUB Part ID^a^	Relevant Characteristics	Source or reference
***E*. *coli* strains**			
BL21(DE3)		F^-^ *ompT gal dcm lon hsdS*_*B*_(r_B_^-^ m_B_^-^) λ(DE3)	[17]
DH10B		F^-^ *mcrA endA1 recA1 galE15 galK16 nupG rpsL* Δ*lacX74* Φ80*lacZ*ΔM15 *araD139* Δ(*ara leu*)7697 Δ*(mrr-hsdRMS-mcrBC*) λ-	[18]
DH5αZ1		*attB*::[*lacI*^q^ P_N25_-*tetR* Sp^R^] F^-^ Δ*(argF-lac)169* Φ80d*lacZ58*(M15) Δ*phoA8 glnX44*(AS) λ^-^ *deoR481 gyrA96*(Nal^R^) *recA1 endA1 thiE1 hsdR17*	[19]
MG1655		F^-^ λ^-^ *rph-1*	[20]
ladd-initial	JPUB_006782	DH5αZ1with pPJ176	This study
op3 final	JPUB_006764	DH5αZ1with with pPJ167	This study
op4 final	JPUB_006766	DH5αZ1with with pPJ168	This study
op5 final	JPUB_006768	DH5αZ1with with pPJ169	This study
op6 final	JPUB_006770	DH5αZ1with with pPJ170	This study
op7 final	JPUB_006772	DH5αZ1with with pPJ171	This study
op8 final	JPUB_006774	DH5αZ1with with pPJ172	This study
op10 final	JPUB_006776	DH5αZ1with with pPJ173	This study
Lyc-no-CrtI	JPUB_006743	MG1655 with pPJ179	This study
Lyc36	JPUB_006744	MG1655 with pPJ177	This study
Lyc07	JPUB_006745	MG1655 with pPJ178	This study
Lyc	JPUB_006810	MG1655 with pLyc	[21]
MBP-3336	JPUB_006784	BL21(DE3) with pPJ180	This study
MBP-3607	JPUB_006786	BL21(DE3) with pPJ181	This study
MBP-CrtI	JPUB_006788	BL21(DE3) with pPJ182	This study
N-His-3336	JPUB_006790	BL21(DE3) with pPJ183	This study
N-His-3607	JPUB_006792	BL21(DE3) with pPJ184	This study
N-His-CrtI	JPUB_006794	BL21(DE3) with pPJ185	This study
C-His-3336	JPUB_006796	BL21(DE3) with pPJ186	This study
C-His-3607	JPUB_006798	BL21(DE3) with pPJ187	This study
C-His-CrtI	JPUB_006800	BL21(DE3) with pPJ188	This study
N-StrepII-3607	JPUB_006806	BL21(DE3) with pPJ219	This study
C-StrepII-3607	JPUB_006808	BL21(DE3) with pPJ220	This study
N-8xHis-StrepII-MBP-3607	JPUB_006802	BL21(DE3) with pPJ217	This study
**Plasmids**			
pFAB217		Km^R^; p15a vector	[22]
pBbA0k		Km^R^; p15a vector	[23]
pBbE0k		Km^R^; ColE1 vector	[23]
pBbE0a_mut		Amp^R^; ColE1 vector; BsaI site removed by mutagenesis	[23]
pET24		Km^R^; T7 promoter	Novagen
pET28a		Km^R^; T7 promoter	Novagen
pET28a-MBP		Km^R^; T7 promoter; maltose-binding protein (MBP) tag sequence	Footnote ^b^
pSKB3-EL3		Km^R^; T7 promoter; 8xHis, StrepII, and MBP tag sequences	Footnote ^c^
pPJ174	JPUB_006779	Km^R^; operon 1 genes (kuste3603, kuste3605, kuste3606) with unique P_tet_ promoter, BCD, and terminator in pFAB217	This study
pPJ175	JPUB_006781	Km^R^; operon 2 genes (kuste3604, kuste3607, kuste3608) with unique P_tet_ promoter, BCD, and terminator in pBbA0k	This study
pPJ176	JPUB_006783	Km^R^; EcoRI- and BamHI-digestion fragment of pPJ174 ligated into EcoRI and BglII sites of pPJ175	This study
pPJ158	JPUB_006747	Km^R^; operon 3 genes (kuste2803, kuste2804) in pBbE0k	This study
pPJ159	JPUB_006749	Km^R^; operon 4 genes (kuste2802, kuste2805) in pBbE0k	This study
pPJ160	JPUB_006751	Amp^R^; operon 5 genes (kuste3340, kuste3350) in pBbE0a_mut	This study
pPJ161	JPUB_006753	Km^R^; operon 6 genes (kuste3346, kuste3348, kuste3349) in pBbE0k	This study
pPJ162	JPUB_006755	Amp^R^; operon 7 genes (kuste3338, kuste3347) in pBbE0a_mut	This study
pPJ163	JPUB_006757	Amp^R^; operon 8 genes (kuste3342, kuste3343) in pBbE0a_mut	This study
pPJ164	JPUB_006759	Amp^R^; operon 9 genes (kuste3345, kuste3351, kuste3352) in pBbE0a_mut	This study
pPJ165	JPUB_006761	Km^R^; operon 10 genes (kuste3336, kuste3339, kuste3341) in pBbE0k	This study
pPJ166	JPUB_006763	Amp^R^; operon 11 genes (kuste3335, kuste3344) in pBbE0a_mut	This study
pPJ149	JPUB_006726	Km^R^; new unique P_tet_ promoter and terminator for operon 3 in pBbA0k	This study
pPJ150	JPUB_006728	Km^R^; new unique P_tet_ promoter and terminator for operon 4 in pBbA0k	This study
pPJ151	JPUB_006730	Km^R^; new unique P_tet_ promoter and terminator for operon 5 in pBbA0k	This study
pPJ152	JPUB_006732	Km^R^; new unique P_tet_ promoter and terminator for operon 6 in pBbA0k	This study
pPJ153	JPUB_006722	Km^R^; new unique P_tet_ promoter and terminator for operon 7 in pBbA0k	This study
pPJ154	JPUB_006734	Km^R^; new unique P_tet_ promoter and terminator for operon 8 in pBbA0k	This study
pPJ146	JPUB_006724	Km^R^; old P_tet_ promoter, BCD, and terminator for operon 9 in pBbA0k	This study
pPJ156	JPUB_006736	Km^R^; new unique P_tet_ promoter and terminator for operon 10 in pBbA0k	This study
pPJ167	JPUB_006765	Km^R^; operon 3 genes in pPJ149	This study
pPJ168	JPUB_006767	Km^R^; operon 4 genes in pPJ150	This study
pPJ169	JPUB_006769	Km^R^; operon 5 genes in pPJ151	This study
pPJ170	JPUB_006771	Km^R^; operon 6 genes in pPJ152	This study
pPJ171	JPUB_006773	Km^R^; operon 7 genes in pPJ153	This study
pPJ172	JPUB_006775	Km^R^; operon 8 genes in pPJ154	This study
pPJ173	JPUB_006777	Km^R^; operon 10 genes in pPJ156	This study
pLyc		Cm^R^; BR322 vector with *araC* repressor; *crtE*, *crtI*, and *crtB* under P_BAD_ promoter	[21]
pPJ179	JPUB_006738	Cm^R^; pLyc with *crtI* gene removed	This study
pPJ177	JPUB_006740	Cm^R^; pLyc with kuste3336 replacing *crtI*	This study
pPJ178	JPUB_006742	Cm^R^; pLyc with kuste3607 replacing *crtI*	This study
pPJ180	JPUB_006785	Km^R^; kuste3336 with N-terminal MBP tag sequence in pET28a-MBP	This study
pPJ181	JPUB_006787	Km^R^; kuste3607 with N-terminal MBP tag sequence in pET28a-MBP	This study
pPJ182	JPUB_006789	Km^R^; *Pantoea agglomerans crtI* with N-terminal MBP tag sequence in pET28a-MBP	This study
pPJ183	JPUB_006791	Km^R^; kuste3336 with N-terminal 6xHis tag sequence in pET28a	This study
pPJ184	JPUB_006793	Km^R^; kuste3607 with N-terminal 6xHis tag sequence in pET28a	This study
pPJ185	JPUB_006795	Km^R^; *Pantoea agglomerans crtI* with N-terminal 6xHis tag sequence in pET28a	This study
pPJ186	JPUB_006797	Km^R^; kuste3336 with C-terminal 6xHis tag sequence in pET24	This study
pPJ187	JPUB_006799	Km^R^; kuste3607 with C-terminal 6xHis tag sequence in pET24	This study
pPJ188	JPUB_006801	Km^R^; *Pantoea agglomerans crtI* with C-terminal 6xHis tag sequence in pET24	This study
pPJ218	JPUB_006805	Km^R^; *E*. *coli acpP* with N-terminal StrepII tag sequence in pET24	This study
pPJ219	JPUB_006807	Km^R^; kuste3607 with N-terminal StrepII tag sequence in pET24	This study
pPJ220	JPUB_006809	Km^R^; kuste3607 with C-terminal StrepII tag sequence in pET24	This study
pPJ217	JPUB_006803	Km^R^; kuste3607 with N-terminal 8xHis, StrepII, and MBP tag sequences in pSKB3-EL3	This study

^a^ Accessible *via* public instance of the JBEI Registry (https://public-registry.jbei.org/folders/222)

^b^ Provided by Andrew Hagen (JBEI).

^c^ Provided by Aindrila Mukhopadhyay (JBEI).

### Synthesized DNA sequences, including candidate ladderane biosynthesis genes from anammox bacteria

All 34 *K*. *stuttgartiensis* genes identified as candidates for the ladderane biosynthesis pathway [11] were codon-optimized for expression in *E*. *coli* using an empirically derived codon usage table [24]. Codon optimization, including restriction site removal, and oligo design (150mers) were performed using GeneDesign [25]. Oligos were pooled for synthesis using acoustic deposition (Labcyte Echo 550), and synthesis was performed at the Joint Genome Institute using a 2-step PCA approach in 2 μL final volume as previously described [26]. PCA products were purified by gel excision and cloned into pENTR (Life Technologies) by In-Fusion cloning (Clontech). Plating and picking were performed using a QPix 400 system (Molecular Devices). Eight colonies per construct were sequence-verified using PACBIO RSII system (Pacific Biosciences). Synthesized gene sequences are listed in S2 Table.

Operon regulatory elements consisted of unique tetracycline-inducible promoters (P_tet_), bicistronic design (BCD) elements, and terminator sequences, which were obtained from BIOFAB [22, 27]. Additional DNA parts were purchased as gBlocks or oligonucleotides from Integrated DNA Technologies (Coralville, IA). Oligonucleotides used for DNA assemblies, with the exception of those used for restriction digest and ligation, were designed using j5 software [28, 29]. Operon composition, DNA assembly templates and primers, and gBlock sequences are listed in S3, S4 and S5 Tables, respectively.

### Operon plasmid assembly

Candidate ladderane biosynthesis genes were divided among 11 synthetic operons based upon putative function (Table 2). The operon 1 plasmid was assembled as follows. The promoter and BCD sequences were PCR-amplified from a gBlock, while gene CDSs, including intergenic RBSs, were PCR-amplified from JGI shuttle plasmids carrying each respective gene. These parts were assembled with a PCR-amplified pFAB217 backbone (p15A ori, *kanR*) using a modified Golden Gate [30] assembly method, which involved BsaI digestion of parts overnight, followed by ligation with a high-concentration ligase for 30 min at room temperature. The operon 2 plasmid was assembled in a similar fashion as operon 1, with the exception that a pBbA0k backbone (p15A ori, *kanR*) [23] was used. The original terminator in the operon 2 plasmid was later replaced with a his[min] terminator by inverse PCR using phosphorylated primers containing the necessary sequence to be added. Each assembly was sequence-verified. Finally, operons 1 and 2 were combined in a single plasmid, pPJ176 (Table 1), by isolating operon 1 through EcoRI and BamHI digestion and inserting the operon 1 fragment into the operon 2 plasmid backbone, which had been digested with EcoRI and BglII.

**Table 2:** Synthetic operons used in this study.

Operon	Gene	Putative Function	Notes
1	kuste3603	ACP (acyl carrier protein)	Operon 1 + Operon 2 genes constitute the second most highly expressed candidate gene cluster in *K*. *stuttgartiensis* and encode some unique products
1	kuste3605	FabF (β-ketoacyl-ACP synthase II)	(see above)
1	kuste3606	non-canonical FabF	(see above)
2	kuste3604	FabZ (β-hydroxyacyl-ACP dehydratase)	(see above)
2	kuste3607	phytoene desaturase	(see above)
2	kuste3608	SAM radical enzyme	(see above)
3	kuste2803	SAM radical enzyme	Operon 3 + Operon 4 genes constitute a complete cluster in *K*. *stuttgartiensis* and encode some unique products
3	kuste2804	FabF	(see above)
4	kuste2802	ACP synthase	(see above)
4	kuste2805	non-canonical FabB (β-ketoacyl-ACP synthase I)	(see above)
5	kuste3340	ACP	Activate fatty acid synthesis intermediates
5	kuste3350	ACP	(see above)
6	kuste3346	FabF	β-ketoacyl-ACP synthase activity
6	kuste3348	non-canonical FabF	(see above)
6	kuste3349	FabF	(see above)
7	kuste3338	SAM methylase	SAM radical reactions are hypothesized to play a role in ladderane cyclization
7	kuste3347	SAM methylase	(see above)
8	kuste3342	SAM radical enyzme	(see above)
8	kuste3343	SAM radical enzyme	(see above)
9	kuste3345	unknown	Function of gene product is unknown
9	kuste3351	conserved hypothetical protein	(see above)
9	kuste3352	unknown	(see above)
10	kuste3336	phytoene desaturase	Catalysis of putative desaturation or reduction reactions
10	kuste3339	FabZ	(see above)
10	kuste3341	FabG	(see above)
11	kuste3335	glycosyltransferase	Function not typically associated with fatty acid synthesis
11	kuste3344	phenylacetyl-CoA ligase	(see above)

**Fig 2:**
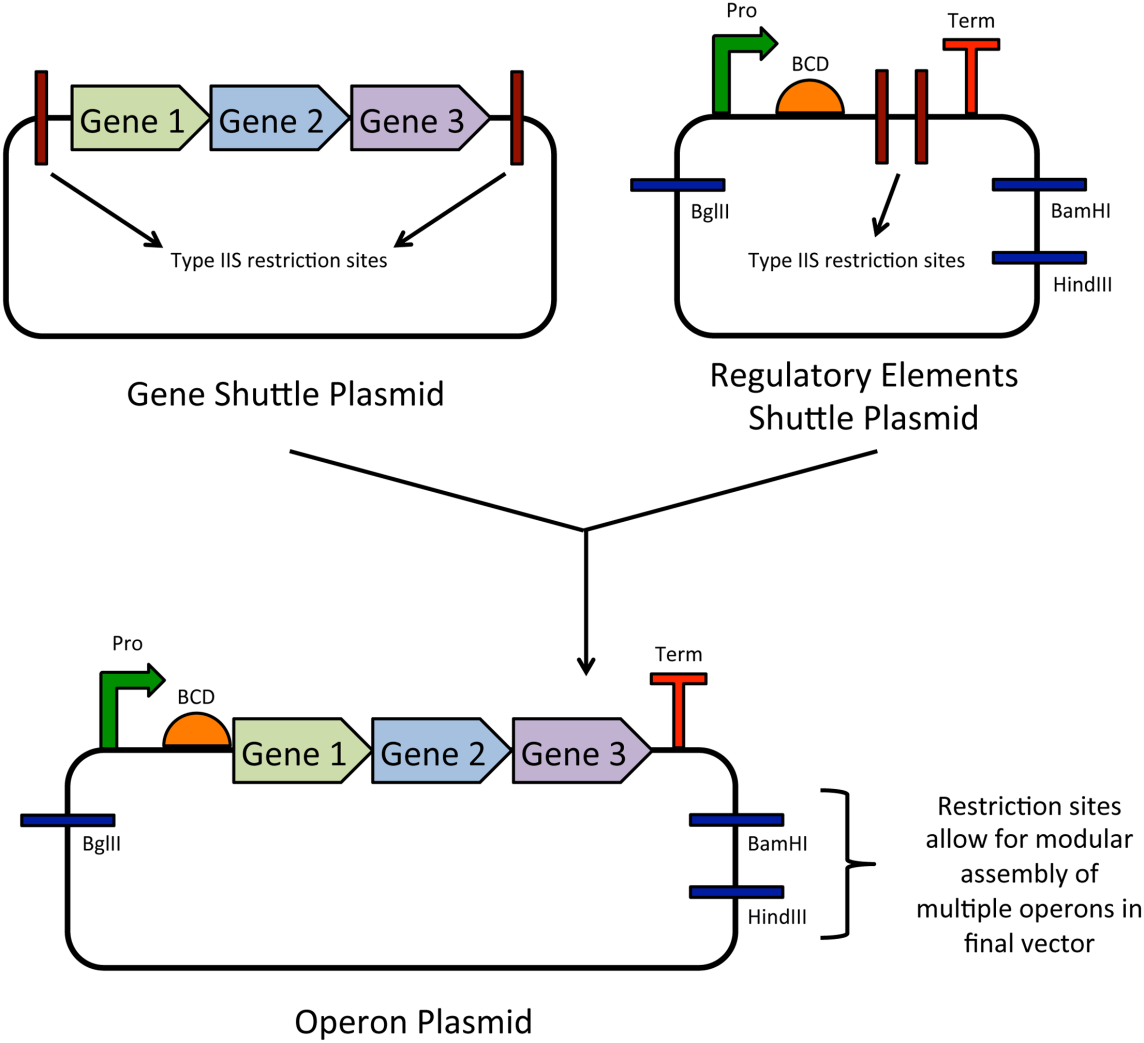
DNA assembly scheme for construction of operons 3–11 (see Table 2 for additional detail). Each operon has a unique P_tet_ promoter, bicistronic design (BCD) element, and terminator chosen from the BIOFAB database. Restriction sites in each final operon plasmid allow for efficient, modular assembly of multiple operons in a final vector, such as a bacterial artificial chromosome or fosmid.

Plasmids carrying completed operons 3–11 were each assembled from two separate shuttle plasmids, one carrying operon genes and the other carrying regulatory elements (P_tet_ promoter, BCD, terminator) (Fig 2). For the operon 3, 4, 6, and 10 gene plasmids, respective genes were PCR-amplified from JGI shuttle plasmids and cloned into a pBbE0k backbone (colE1 ori, *kanR*), which had been digested with BamHI and XhoI, through Gibson assembly [31]. The operon 5, 7, 8, 9, and 11 gene plasmids were assembled in a similar fashion, with the exception that the backbone was derived from BamHI and XhoI digestion of pBbE0a_mut, (colE1 ori, *ampR*, BsaI site removed through mutagenesis). For operons containing more than two genes (6, 9, 10), two-step assemblies were conducted to assemble the complete gene plasmids. Each assembly reaction was sequence-verified.

The regulatory-element shuttle plasmid for each operon consisted of promoter, BCD, and terminator sequences that were PCR-amplified from gBlocks and assembled in a PCR-amplified pBbA0k backbone, using the CPEC technique [32]. Shuttle plasmids for operons 5, 7, 8, and 9 required an additional assembly step because the complete sequence for promoter or terminator could not be included in the initial CPEC assembly primers, due to possible mispriming. The missing sequences for each of these plasmids were introduced by inverse PCR using phosphorylated primers. Each assembly reaction was sequence-verified. The gene and regulatory elements shuttle plasmids for each operon had complementary Type IIS restriction sites (S4 Table). These sites were used in Golden Gate assemblies to insert each set of operon genes into the respective regulatory elements plasmids, yielding the final operon plasmids.

### Operon promoter replacement

The promoters for operons 3–11 were replaced with stronger variants from the BIOFAB registry that were previously characterized [22]. Each of the new promoters was introduced into its respective regulatory elements shuttle plasmid through one-part CPEC assemblies. To simplify analysis of protein expression, BCDs were removed during assembly. The new promoters were combined with the respective gene sets as described above to produce new operons 3–11. Sequences for the new promoters are listed in S3 Table.

### DNA assembly for protein expression of putative desaturases

Vectors for expression of each C-terminal 6xHis-tagged desaturase were prepared by PCR-amplifying genes and inserting into an NdeI- and XhoI-digested pET24 vector backbone (Novagen Biosciences, Madison, WI) through Gibson assembly. The kuste3336 and kuste3607 genes were amplified from JGI shuttle plasmids, whereas the *Pantoea agglomerans crtI* gene was PCR-amplified from the previously described pLyc plasmid [21] (Table 1). Gibson assemblies were also performed to introduce the kuste3607 gene into each of two separate pET24 backbones, which were PCR-amplified from pET24-N-StrepII-EcAcpP (pPJ218; Table 1), to give constructs expressing either N- or C-terminal StrepII-tagged proteins. N-6xHis- and N-6xHis-MBP-tagged expression constructs were prepared by PCR-amplifying kuste3607 from its JGI shuttle plasmid, digesting with NdeI and XhoI, and ligating into either digested pET28 or pET28a-MBP (JBEI ICE Part ID JBx_014631), respectively. The kuste3607 gene was also inserted into pSKB3-EL3 (JBEI-7594) through Gibson assembly to construct a vector expressing N-8xHis-StrepII-MBP-3607, which has a tobacco etch virus (TEV) protease recognition site.

The pLyc-no-CrtI plasmid (pPJ179; Table 1) was constructed through a one-part CPEC assembly [32] that removed the *crtI* gene from pLyc. pLyc-3336 and pLyc-3607 plasmids (pPJ177 and pPJ178; Table 1) were prepared through Gibson assemblies to replace the pLyc *crtI* gene with either the kuste3336 or kuste3607 gene, respectively. Protein sequences are listed in S6 Table.

### Cell growth and fatty acid production

Anaerobic fatty acid production in *E*. *coli* DH5αZ1 transformed with pPJ176 (operons 1 and 2; Table 1) was compared to that of DH5αZ1 carrying the empty pBbA0k vector. For each strain, a 250-mL serum bottle containing 200 mL of EZ Rich medium (Teknova, Hollister, CA) supplemented with 0.2% glucose and 50 μg/mL kanamycin was inoculated from an overnight culture to a starting OD_600_ of 0.005 and sealed with a butyl rubber stopper. The culture was incubated at 37°C shaking at 200 rpm until the OD_600_ reached ~0.4, at which point gene expression was induced by addition of 200 nM anhydrotetracycline (ATc) under anaerobic conditions. Anaerobic growth was continued at 37°C overnight. The next day, cells were harvested in 30-mL high-strength glass centrifuge tubes and supernatant was decanted. The cell pellet was flash-frozen in liquid nitrogen and lyophilized overnight in a Labconco lyophilizer (Kansas City, MO). Biomass was stored at room temperature until fatty acid extraction.

The remaining operon strains were individually tested in the same manner, with the exception that cultures were grown in 125-mL serum bottles containing 100 mL media, and 3 mM KNO_3_ was added under anaerobic conditions at the time of induction, where noted, to obtain higher cell density.

### Extraction and GC/MS analysis of fatty acids

All solvents used were pesticide-residue-analysis grade and all glassware was washed with ultrapure acetone. The extraction method was modified from that described by Sinninghe Damsté *et al*. [33] Briefly, 6 mL MeOH was added to lyophilized biomass in a high-strength glass centrifuge tube, which was then vortexed and sonicated in an ice water bath for 10 min, followed by centrifugation at 5000 x *g* for 5 min at 20°C and collection of solvent in a 40-mL pre-cleaned glass vial. The extraction process was repeated once with 6 mL of MeOH:CH_2_Cl_2_ (1:1) and three times with 6 mL CH_2_Cl_2_, resulting in 30 mL extract. The extract was evaporated to dryness using an R-210 rotary evaporator (Buchi, Flawil, Switzerland). The residue was reconstituted in ~2 mL CH_2_Cl_2_, transferred to a 10-mL Reacti-Vial, and evaporated to ~100 μL under an ultra high purity nitrogen gas stream. Extracts were then derivatized with ethereal diazomethane prepared in an Aldrich diazomethane-generator (Sigma-Aldrich), followed by reconstitution in 100 μL CH_2_Cl_2_.

GC-MS analyses were performed with a model 7890A GC (Agilent, Santa Clara, CA) with a DB-5 fused silica capillary column (30-m length, 0.25-mm inner diameter, 0.25-μm film thickness; J & W Scientific) coupled to an HP 5975C series quadrupole mass spectrometer. One-μL injections were performed by a model 7683B autosampler. The GC oven was programmed from 40°C (held for 2 min) to 130°C at 15°C/min, then to 300°C at 5°C/min and held for 10 min; the injection port temperature was 250°C, and the transfer line temperature was 280°C. The carrier gas, ultra-high-purity helium, flowed at a constant rate of 1 mL/min. Injections were splitless, with the split turned on after 0.5 min. The extraction and GC/MS analysis methods were validated by the detection of ladderane fatty acids (e.g., a C_20_ [3]-ladderane fatty acid derivatized as a methyl ester) from anammox culture biomass samples graciously provided by Barth F. Smets of the Technical University of Denmark.

### Expression and purification of kuste3607

An overnight culture of *E*. *coli* BL21(DE3) expressing N-8xHis-StrepII-MBP-3607 (Table 1) was used to inoculate two 2-L baffled flasks, each containing 1 liter of lysogeny broth (LB) supplemented with 50 μg/mL kanamycin. Cultures were grown at 37°C until the OD_600_ reached ~0.5, at which point protein expression was induced by addition of 50 μM IPTG and growth continued at 18°C overnight. Cells were harvested the next day and stored at -80°C until further processing. The pellet was resuspended in 100 mL of Buffer L consisting of 50 mM sodium phosphate (pH 7.5), 500 mM NaCl, 10% glycerol, 1 mM DTT, 0.2 mg/mL lysozyme, 10 mM MgCl_2_, 10 μg/mL DNase I, and two Pierce Protease Inhibitor Mini Tablets (Thermo Scientific, Wilmington, DE). Cells were lysed using an EmulsiFlex-C3 high-pressure homogenizer (Avestin, Ottawa, ON, Canada), followed by centrifugation of lysate at 15000 x *g* for 30 min at 4°C. Soluble lysate was aspirated and subjected to purification on an ÄKTAexplorer FPLC system equipped with a 5-mL StrepTrap HP column (GE Healthcare Life Sciences, Marlborough, MA). Following injection, protein was washed with Buffer A [50 mM sodium phosphate (pH 7.5), 500 mM NaCl, 10% glycerol, 1 mM DTT] and eluted with 6 column volumes (CV) of Buffer B (A + 2.5 mM desthiobiotin). Elution samples were analyzed by SDS-PAGE and fractions containing eluted 3607 were pooled. Approximately 5 mg of protein was obtained in this fashion. 0.1% Igepal CA-630 was added to the protein, which was then concentrated to ~2 mL with a 30-kDa MWCO concentrator. Recombinant TEV protease was added to the protein at a ratio of 1:100 protease:protein and incubated at 4°C overnight to cleave fusion tags from the 3607 enzyme. The cleaved protein was used for assays the following day.

### Cell growth for lycopene production

Overnight cultures of *E*. *coli* MG1655 expressing pPJ179, pPJ177, pPJ178, or pLyc were used to inoculate 50 mL LB supplemented with 30 μg/mL chloramphenicol. Cultures were grown at 37°C until the OD_600_ reached ~0.5, at which point 10 μM L-arabinose was added and growth continued at 18°C overnight. The next day, 20 OD x mL of culture was harvested and frozen for proteomic analysis, while the remainder pelleted in high-strength glass centrifuge tubes, which were stored at -80°C until product extraction.

### Lycopene extraction and HPLC analysis

To each frozen cell pellet, 1 mL MeOH and 4 mL hexane were added. Samples were vortexed and sonicated in an ice-water bath for 15 min, followed by incubation at room temperature for 10 min and centrifugation at 5000 x *g* for 15 min at 20°C to separate the organic and aqueous phases. The hexane layer was transferred to a 10-mL Reacti-Vial and concentrated to 50 μL under a gentle nitrogen gas stream.

Extracts were subjected to HPLC analysis using an Agilent 1200 series HPLC system, with a 3 μm, 250-mm x 2.1-mm reverse-phase Inertsil ODS-3 column (GL Sciences, Tokyo, Japan) as previously described [34], with the exception that lycopene was detected at 470 nm.

### Reverse transcription quantitative polymerase chain reaction (RT-qPCR) analysis

For RT-qPCR analysis of gene expression from operons 1 and 2, 15-mL samples from induced and non-induced cultures were collected and transferred to 50-mL tubes containing 1.9 mL of 5% phenol in ethanol. The cell mixture was incubated on ice for 10 min and then centrifuged at 1600 x *g* for 10 min at 4°C. Supernatant was decanted and the pellet flash-frozen in liquid nitrogen for storage at -80°C until further processing. mRNA was extracted with a RNeasy Mini Kit (Qiagen, Valencia, CA). Qiagen DNaseI was used for on-column DNA digestion and the Turbo DNA-free Kit (Thermo Fisher Scientific, Waltham, MA) for a second DNase treatment after mRNA purification. RNA concentration was quantified with a NanoDrop 1000 spectrophotometer (Thermo Scientific). cDNA was prepared from 4 μg RNA using the SMARTScribe Reverse Transcriptase Kit (Clontech, Mountain View, CA). qPCR was performed with 4 μL of tenfold-diluted cDNA as template and SsoAdvanced SYBR Green Supermix (Bio-Rad, Hercules, CA) on a StepOnePlus Real-Time PCR System (Applied Biosystems, Foster City, CA). qPCR primers were designed with the web-based IDT PrimerQuest tool. The *E*. *coli hcaT* gene was used as an endogenous control [35] and standards consisted of pPJ176 at 0.005, 0.05, 0.5, 5, and 50 ng. Analyses were run in triplicate.

### Shotgun proteomics

For analysis of protein expression from ladd-initial, 25 mL of cells were harvested at the end of growth and lysed with Thermo Scientific B-PER reagent at 4-mL / gram cell paste. Lysate was buffer-exchanged to 100 mM NH_4_HCO_3_ (AMBIC) by three rounds of concentration and dilution with a 3-kDa MWCO concentrator. Samples were then processed as previously described [36].

Analyses of samples from strains individually expressing operons 3–8 and 10 were conducted as for ladd-initial, except 20 OD x mL of cells were used and lysate was sonicated using a Qsonica sonicator (Newtown, CT) equipped with a microtip (5 sec on, 5 sec off, 25 sec processing time, power = 1). Lysate was then clarified by centrifugation at 15000 x *g* for 5 min at 4°C in a microcentrifuge. Soluble lysate was collected and buffer-exchanged to 100 mM AMBIC, while pelleted material was washed three times with tenfold-diluted B-PER reagent. Both soluble and pellet samples were processed for proteomic analysis as previously described [37]. Briefly, the proteins were extracted by chloroform/methanol precipitation and resuspended in 100 mM AMBIC with 20% acetonitrile. The proteins were reduced with tris(2-carboxyethyl)phosphine (TCEP) for 30 min, followed by incubation with iodoacetamide (IAA; 10 mM final) for 30 min in the dark, and overnight digestion with MS-grade trypsin (1:50 w/w trypsin:protein) at 37°C. Samples were analyzed on an Agilent 1290 UHPLC—6550 QTOF liquid chromatography mass spectrometer (LC-MS/MS; Agilent Technologies) system, with previously described operating parameters [37]. Peptides were separated on a Sigma-Aldrich Ascentis Express Peptide ES-C18 column (2.1-mm x 100-mm, 2.7- μm particle size, operated at 60°C) at a flow rate of 0.4 mL/min. The chromatography gradient conditions were as follows: from the initial starting condition [95% buffer A (100% water, 0.1% formic acid) and 5% buffer B (100% acetonitrile, 0.1% formic acid)] the buffer B composition was increased to 35% over 30 min; then buffer B was increased to 80% over 3 min and held for 7 min, followed by a ramp back down to 5% B over 1 min where it was held for 6 min to re-equilibrate the column to original conditions. Data were analyzed with the Mascot search engine version 2.3.02 (Matrix Science) and filtered and validated using Scaffold v4.3.0 (Proteome Software Inc.), as previously described [37].

## Results and Discussion

### Gene synthesis and design of synthetic operons

**Fig 3:**
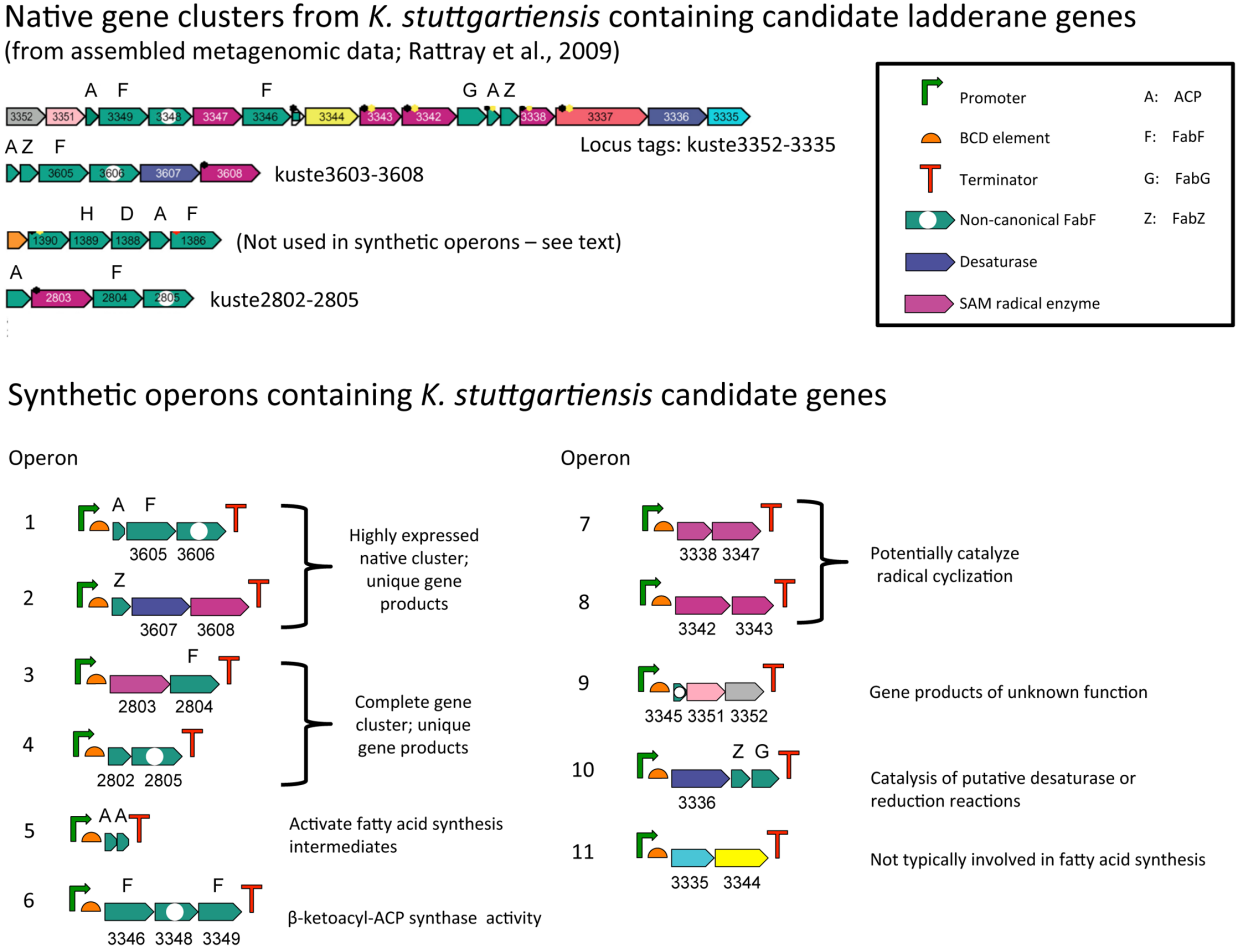
Candidate ladderane synthesis genes from *Kuenenia stuttgartiensis* and synthetic operon design. (Top) Candidate genes identified by Rattray et al. [11] in native gene clusters with locus tags (adapted from [11]). (Bottom) Synthetic operons designed in this study containing candidate genes grouped by putative function or native gene clusters. Locus tags (without the kuste prefix) are shown for most genes in the synthetic operons; more details are given in Table 2.

All 34 *K*. *stuttgartiensis* genes that were previously identified as potential candidates for the ladderane biosynthetic pathway [11] were codon-optimized for expression in *E*. *coli* and synthesized by JGI (S2 Table). To elucidate which of the candidate genes are involved in ladderane production, genes were grouped together in synthetic operons based on putative function (Fig 3, Table 2). This approach supports rational and efficient identification of gene function: changes in fatty acid profiles can be attributed to a putative function (operon), and then to specific gene(s) within the operon. To regulate gene expression at different levels in separate operons and prevent possible homologous recombination within large vectors, unique promoters, translation initiation (bicistronic design, BCD) elements, and terminators were chosen for each operon. Sequences for each of these parts were obtained from the BIOFAB database, which includes characterization data for each part [22, 27]. Previously reported metatranscriptome data [38] were used to select P_tet_ promoters of various relative strengths for each operon in order to simulate native gene expression levels in *K*. *stuttgartiensis* (native expression levels are presented in S7 Table). The P_tet_ system has the beneficial characteristics of low “leaky” expression and low sensitivity to catabolite repression as compared to other inducible promoter systems [23]. BCD elements were used to prevent premature translation termination caused by RNA secondary structure formation. Operons 1 and 2 were combined to form pPJ176 (Table 1), which contains genes kuste3603-3608. This cluster was the focus of preliminary cell growth and fatty acid analyses, as the encoded genes are from the second most highly expressed of the candidate gene clusters after the canonical type II fatty acid synthesis genes kustd1386-1391 (S7 Table). Moreover, the kuste3603-3608 genes encode unique putative enzymes, such as a non-canonical FabF, a desaturase, and a SAM radical enzyme [11] (Table 2). Each of the final operons 3–11 were assembled from two separately constructed shuttle vectors, one carrying genes and the other carrying regulatory element sequences (Fig 2). This approach aids future replacement of promoters, BCDs, or terminators, as sequence changes can be made in the regulatory element shuttle plasmid without affecting gene sequences. Each of the synthetic operon plasmids also contains restriction sites that allow for efficient, modular assembly of multiple operons in different combinations in a final vector, such as a bacterial artificial chromosome or fosmid.

The kustd1391-1386 cluster consists of putative *rpmF*, *plsX*, *fabH*, *fabD*, *acp*, and *fabF* genes (Fig 3). These genes display homology and synteny to the *E*. *coli rpmF*-*fabF* gene cluster involved in canonical type II fatty acid biosynthesis, with the exception that the *K*. *stuttgartiensis fabG* (kuste3341) is located within a separate gene cluster. Because of the homology between the *K*. *stuttgartiensis* kustd gene cluster and *E*. *coli* fatty acid biosynthesis genes, the kustd gene cluster was not included in the synthetic operon design in order to limit the number of operon combinations to be tested and simplify downstream analyses. The kuste3337 gene, encoding a putative membrane protein of unknown function, was also excluded from operon design. Potential roles for the kustd1386-1391 and kuste3337 genes in ladderane production cannot be entirely ruled out, as the pathway may involve an unconventional mode of fatty acid synthesis. These genes can be included in future synthetic operon designs, following the procedure described above.

### Analysis of fatty acids and gene expression in operon strains

**Fig 4:**
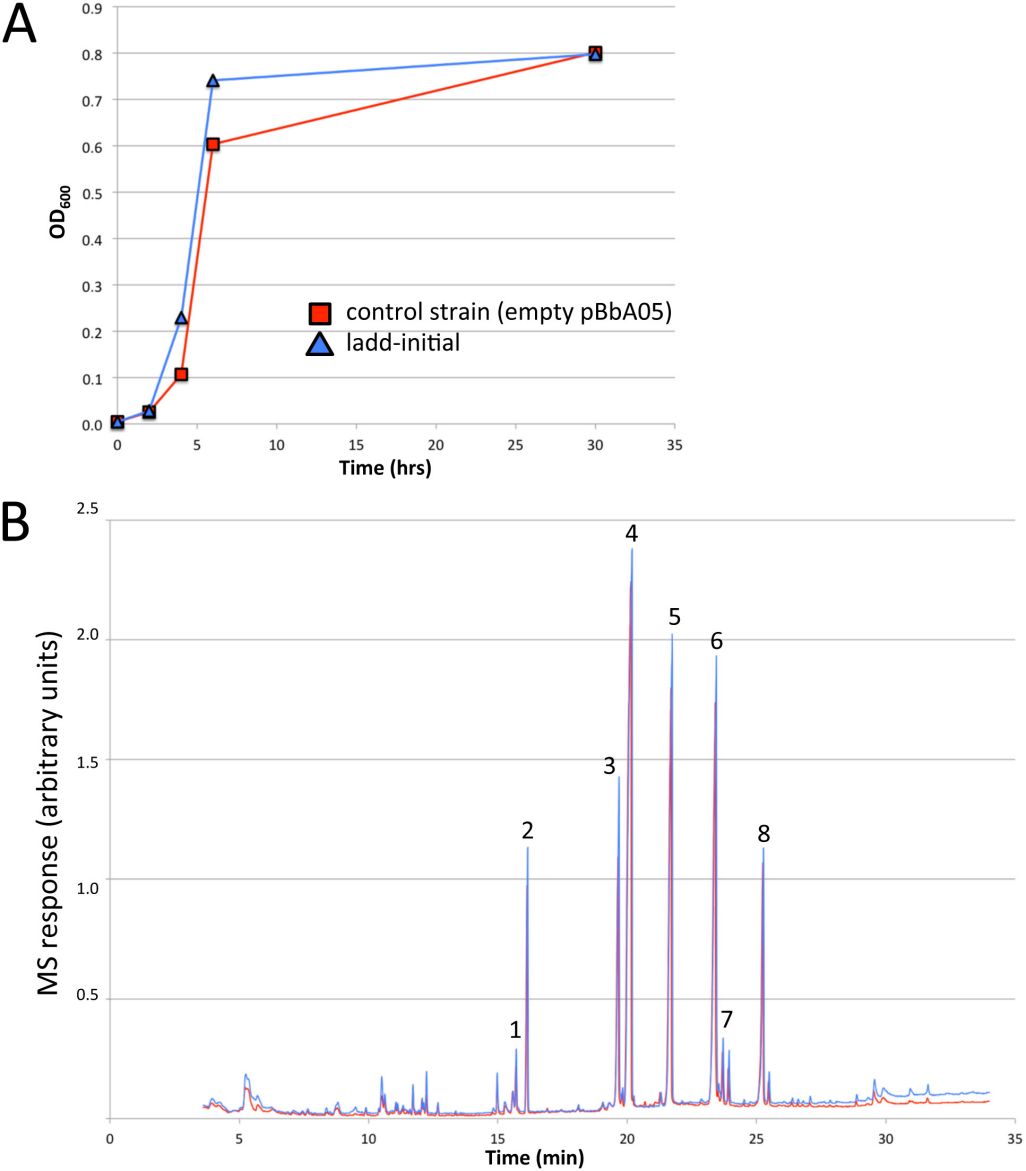
Growth and fatty acid profiles for strain expressing operons 1 and 2 and control strain. (A) Growth curve of ladd-initial and control strains. (B) GC/MS total ion chromatograms (TIC) of fatty acids extracted from ladd-initial and control strains post-cultivation and subjected to methyl ester derivatization. The most prominent fatty acid methyl esters are labeled with numbers: 1, C14:1; 2, C14:0; 3, C16:1; 4, C16:0; 5, C17 cyclopropane fatty acid (CFA); 6, C18:1; 7, C18:0; 8, C19 CFA.

For preliminary analyses, *E*. *coli* DH5αZ1 (containing a chromosomal copy of the *tetR* repressor) [19] carrying pPJ176 (strain ladd-initial; Table 1) was grown under anaerobic conditions to determine whether expression of the kuste3603-3608 genes would lead to changes in fatty acid profile, namely the production of ladderanes or postulated polyunsaturated fatty acid intermediates. The control strain consisted of DH5αZ1 carrying an empty pBbA0k vector. Cell growth rate and final OD_600_ were similar in both strains (Fig 4A). A detailed inspection of GC/MS spectra from both strains did not show any substantial differences in fatty acid profile or the presence of ladderanes or ladderane intermediates (Fig 4B). To determine whether the absence of novel fatty acids was due to impaired gene expression, RT-qPCR was performed to analyze candidate gene transcription. This analysis indicated that each of the kuste3603-3608 genes was transcribed (S1 Fig). Additionally, shotgun proteomic analysis detected each of the expected proteins in whole lysate samples (S8 Table). These combined analyses suggest that the absence of detected products is not due to poor gene expression, but rather that kuste3603-3608 may not be necessary or sufficient for ladderane production.

Before testing various operon combinations, each of operons 3–8 and 10 was individually tested to analyze gene expression and any possible changes in fatty acid profile. Operons 9 and 11 were not tested at this point because the respective genes encode products of unknown function or enzymes that are not typically involved in fatty acid biosynthesis (Fig 3, Table 2). None of the individual operon strains led to ladderane or polyunsaturated fatty acid production, or other novel compounds. When samples of each strain were analyzed through shotgun proteomics, none of the expected proteins were detected (data not shown), suggesting the possibility that the promoters were of insufficient strength to yield detectable levels of protein expression. Each of the P_tet_ promoters for operons 3–11 was thus changed to a stronger variant, based on BIOFAB characterization data [22] (Table 3). Additionally, the BCD elements were removed to preclude these sequences as a confounding factor. New versions of operons 3–8 and 10 (Table 1; op3 final–op8 final; op10 final) were individually tested for gene expression and fatty acid profile changes. No new fatty acid products were observed through GC/MS analysis. The new promoters did lead to improved gene expression: all expected proteins were detected through shotgun proteomic analysis, with the exception of kuste3342, 3343, and 3350 (Table 3). However, the detected proteins were only found in the pellet fractions of lysate samples, except for kuste2802 and kuste3340 proteins, which were detectable at low levels in soluble lysate samples (Table 3). The lack of detection of kuste3350 protein is expected, as the gene encodes a putative, 66-amino acid acyl carrier protein that would be difficult to detect through trypsinization and LC/MS/MS analysis. It is unknown why the kuste3342 and kuste3343 gene products, putative SAM radical, iron-sulfur enzymes, were not detected. One possibility is that inefficient iron-sulfur cluster assembly may have led to degradation or inefficient protein expression [39–41]. The proteomics results raise another possibility, that the lack of detected ladderanes or intermediates is the result of candidate protein insolubility that may arise from inefficient protein folding. Protein insolubility could be addressed by using weaker ribosome binding sites (RBSs) or further optimization of promoter sequences or culture and induction conditions. It is also possible that *E*. *coli* is not an optimal host for heterologous expression of anammox genes, in which case another host could be used for future studies. Nevertheless, the operon assembly scheme and gene expression results provide an efficient system to analyze the effects of candidate gene expression on fatty acid profile.

**Table 3:** Designed operon promoter strength and actual expression based on shotgun proteomic analysis^a^.

Operon	Original Promoter Strength^b^	New Promoter Strength^b^	Gene	% of total MS spectra	Rank^c^
3	25	93	kuste2803	0.16	76
3	^d^	^d^	kuste2804	0.25	38
4	26	100	kuste2802	0.14	91
4	^d^	^d^	kuste2805	0.6	6
5	20	90	kuste3340	0.04	282
5	^d^	^d^	kuste3350	ND^e^	ND
6	13	86	kuste3346	0.57	13
6	^d^	^d^	kuste3348	0.67	8
6	^d^	^d^	kuste3349	0.94	3
7	4	82	kuste3338	0.33	26
7	^d^	^d^	kuste3347	0.45	14
8	7	83	kuste3342	ND	ND
8	^d^	^d^	kuste3343	ND	ND
10	19	89	kuste3336	0.8	3
10	^d^	^d^	kuste3339	0.27	38
10	^d^	^d^	kuste3341	0.37	24

^a^ Samples consist of pellet material after lysate clarification. With the exception of kuste2803 and kuste3340, no target proteins were detected in soluble lysate samples.

^b^ Based on strengths of corresponding constitutive promoter sequences from BIOFAB database, relative to new operon 4 promoter.

^c^ Rank among 740 total detected proteins, based on percentage of total spectra.

^d^ Same as above value for this operon.

^e^ ND, not detected.

### Analysis of *in vivo* lycopene production by putative ladderane desaturases

**Fig 5:**
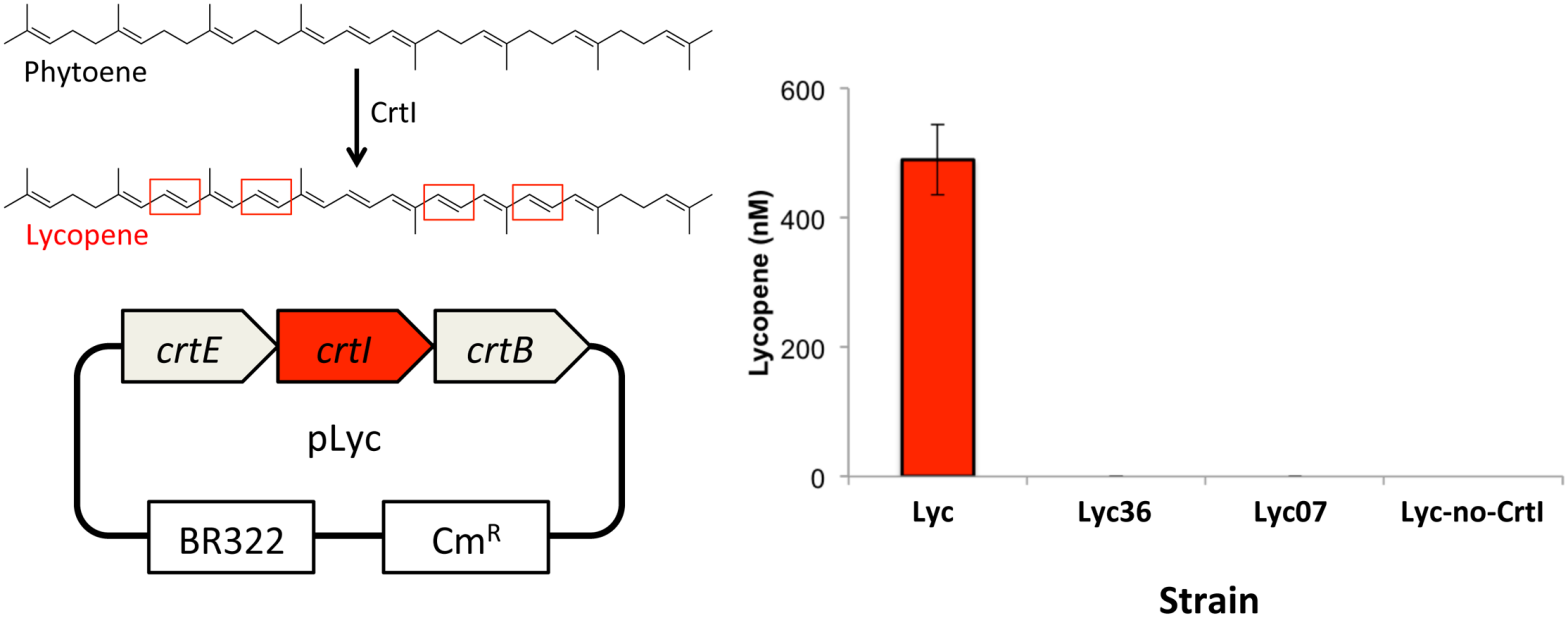
*In vivo* tests of function in putative phytoene desaturases from *K*. *stuttgartiensis* (kuste3336 and kuste3607). (Left) Phytoene desaturation to lycopene catalyzed by CrtI and schematic of the pLyc vector. (Right) Lycopene production in *E*. *coli* MG1655 strains (from left to right): Lyc (positive control), Lyc36 (*crtI* in pLyc replaced with kuste3336), Lyc07 (*crtI* in pLyc replaced with kuste3607), and Lyc-no-CrtI (negative control with *crtI* gene removed) (see Table 1 for details on strains).

The putative desaturase genes were chosen for further analyses because the encoded enzymes could potentially catalyze unique fatty acid modification activity, and are hypothesized to play a crucial role in ladderane synthesis, namely, formation of polyunsaturated fatty acids. The pool of candidate genes includes two putative desaturases, kuste3336 and kuste3607. Both genes are annotated as encoding phytoene desaturases and display 31 and 33% amino acid sequence identity to the lycopene-forming phytoene desaturase (CrtI) from *Methyloglobulus morosus* KoM1, respectively. To test whether these gene products catalyze phytoene desaturase activity, the *crtI* gene in the pLyc vector was separately replaced with kuste3336 and kuste3607 (pPJ177 and pPJ178, respectively, in Table 1). pLyc encodes all the necessary genes to convert farnesyl pyrophosphate to lycopene (including the phytoene desaturase step) [21]. Separate *E*. *coli* MG1655 strains expressing pPJ177 and pPJ178 (Lyc36 and Lyc07, respectively; Table 1) were grown for lycopene production analyses in comparison to a pLyc (no change to *crtI*) strain and a control strain with the *crtI* gene removed (Lyc-no-CrtI; Table 1). HPLC analyses of extracts from each strain indicated that the Lyc36 and Lyc07 strains did not produce lycopene (Fig 5). These results suggest that, despite moderate sequence similarity, the kuste3336 and kuste3607 gene products do not display CrtI-like activity, and may instead display novel functions or substrate specificities, possibly playing a role as fatty acid desaturases in the hypothesized ladderane biosynthetic pathway [11].

### Optimization of purification of putative ladderane-related desaturases

**Fig 6:**
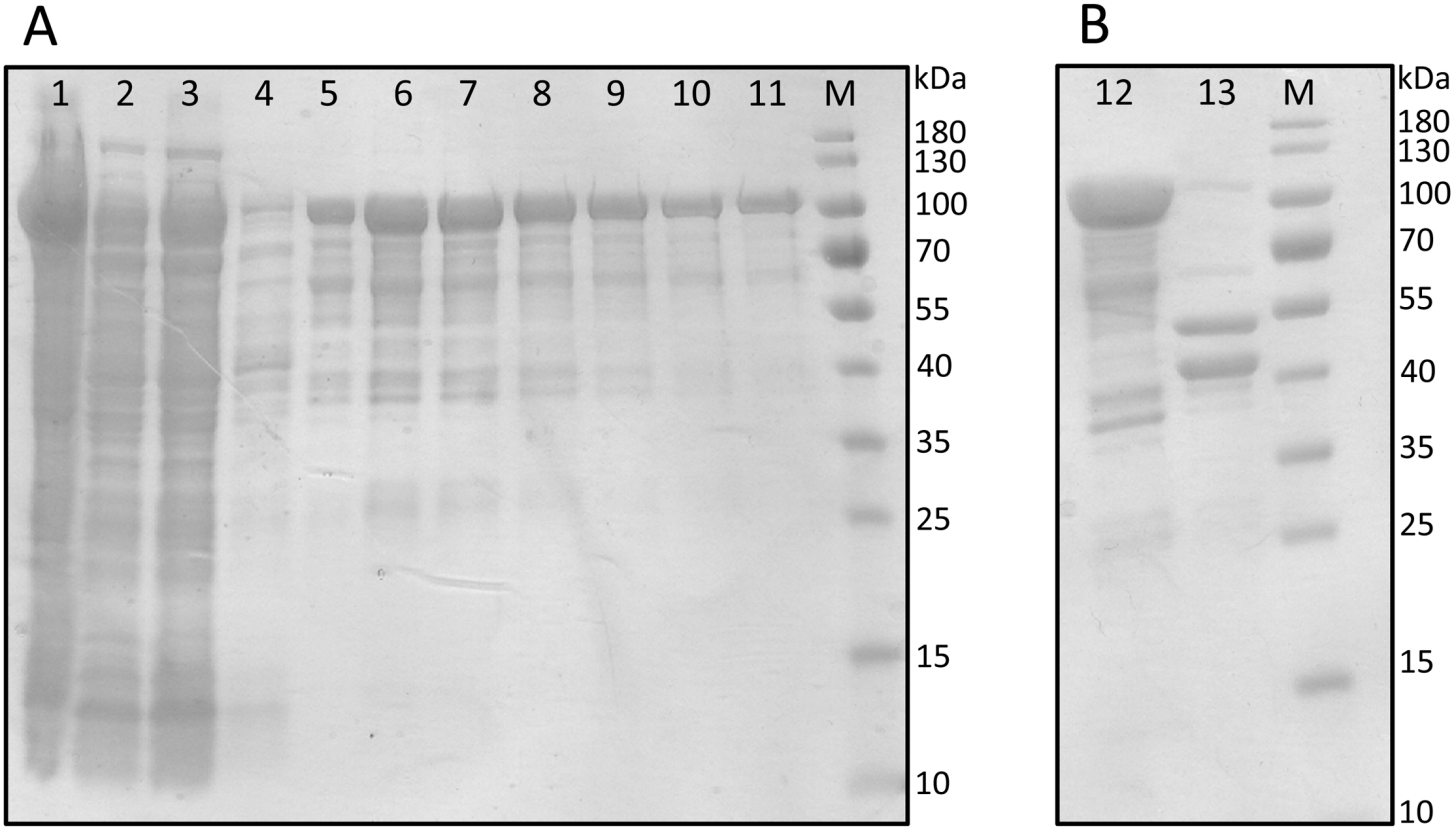
SDS-PAGE analyses of 8xHis-StrepII-MBP-3607 purification (A) and tobacco etch virus (TEV) protease cleavage (B). Lanes: M, molecular mass markers; Lane 1, pellet material after lysate clarification; Lane 2, soluble lysate; Lane 3, StrepTrap column flowthrough; Lane 4, protein washed from column; Lanes 5–11, protein elution fractions; Lane 12, pooled elution fractions; Lane 13; protein after digestion with TEV protease. The expected molecular masses of 8xHis-StrepII-MBP-3607, tag-free 3607 protein, and MBP are 100, 56, and 44 kDa, respectively.

In addition to *in vivo* analyses of lycopene production, soluble expression of the putative desaturases was desired for in-depth *in vitro* assays. Based on the previously described structural work on the CrtI phytoene desaturase from *Pantoea ananatis* [42], plasmids encoding the kuste3336 and kuste3607 genes with C-terminal 6xHis tags were assembled. The kuste3336 protein displayed poor expression and, while kuste3607 expressed, the majority of the expressed protein was insoluble. Optimization of growth, induction, and lysis conditions did not appreciably improve the solubility of the C-6xHis-tagged protein. Plasmids encoding kuste3607 with different tags were then assembled, including N-6xHis-, N-StrepII-, C-StrepII-, and MBP-tagged variants (Table 1). The most soluble version of kuste3607 was obtained as an N-8xHis-StrepII-MBP-tagged protein, which could be purified through StrepTactin-based affinity chromatography and separated from the tags by cleavage with recombinant TEV protease (Fig 6). Since kuste3607 appears not to be a phytoene desaturase based on *in vivo* studies, but may serve some as-yet unidentified role in ladderane biosynthesis, the ability to express it in soluble form may facilitate future experiments of its actual function.

Less extensive expression studies were performed with the SAM radical proteins. Some soluble expression was observed for the C-terminally His-tagged versions of kuste2803 and kuste3608 (strains JPUB_006827 and JPUB_006833, respectively; S6 Table).

## Conclusions

This study describes an efficient means of assembly of 34 synthesized, codon-optimized candidate ladderane biosynthesis genes in synthetic operons that allows for changes to regulatory element sequences, as well as modular assembly of multiple operons for simultaneous heterologous expression in *E*. *coli* (or potentially other microbial hosts). Initial analyses of gene expression indicate that protein insolubility may represent a challenge for fatty acid profile studies, but this can potentially be addressed through further optimization of promoter and RBS sequences or through changes in culture conditions. The lycopene production assay results suggest that the putative desaturases encoded by kuste3336 and kuste3607 do not possess CrtI-like activity, despite sequence similarity among the enzymes and their annotated function. The desaturases may instead display novel functions that are crucial for ladderane synthesis, namely desaturation of fatty acids to form intermediates that are cyclized through downstream radical reactions. The purification scheme for the kuste3607 desaturase described above can be used to prepare soluble protein for in-depth assays aimed at determining the function and substrate specificity of the desaturase. This study is a first step toward elucidating the unique biosynthetic pathway for production of ladderane fatty acids. We invite the scientific community to take advantage of the synthetic biology resources and experimental results developed in this study to elucidate the biosynthetic pathway that produces unique and intriguing ladderane lipids.

## Supporting Information

S1 Fig Fold-increase in mRNA levels from genes in operons 1 and 2 upon induction. Fold-increase is given as relative quantification (RQ) value, obtained by the ΔΔC_T_ method. Bars represent the range between the minimum and maximum RQ values.
(TIF)

S1 Table Comparison of volumetric energy densities for fused cyclobutanes and petroleum-derived and renewable jet fuels. (PDF)

S2 Table JGI-synthesized *Kuenenia stuttgartiensis* genes (XLSX)

S3 Table Synthetic operon composition. (XLSX)

S4 Table Templates and PCR primers used for assemblies. (XLSX)

S5 Table gBlock sequences. (XLSX)

S6 Table Sequences of proteins in expression studies. (XLSX)

S7 Table Relative expression levels for ladderane candidate genes based on transcriptional data presented by Kartal et al. [37]. (XLSX)

S8 Table Shotgun proteomics analysis of operons 1 and 2. (XLSX)
