# Supplementary figures and images for: Investigation of Proposed Ladderane Biosynthetic Genes from Anammox Bacteria by Heterologous Expression in E. coli
Source: PLoS One. 2016 Mar 14;11(3):e0151087. doi: 10.1371/journal.pone.0151087 (PMC4790861; doi:10.1371/journal.pone.0151087)

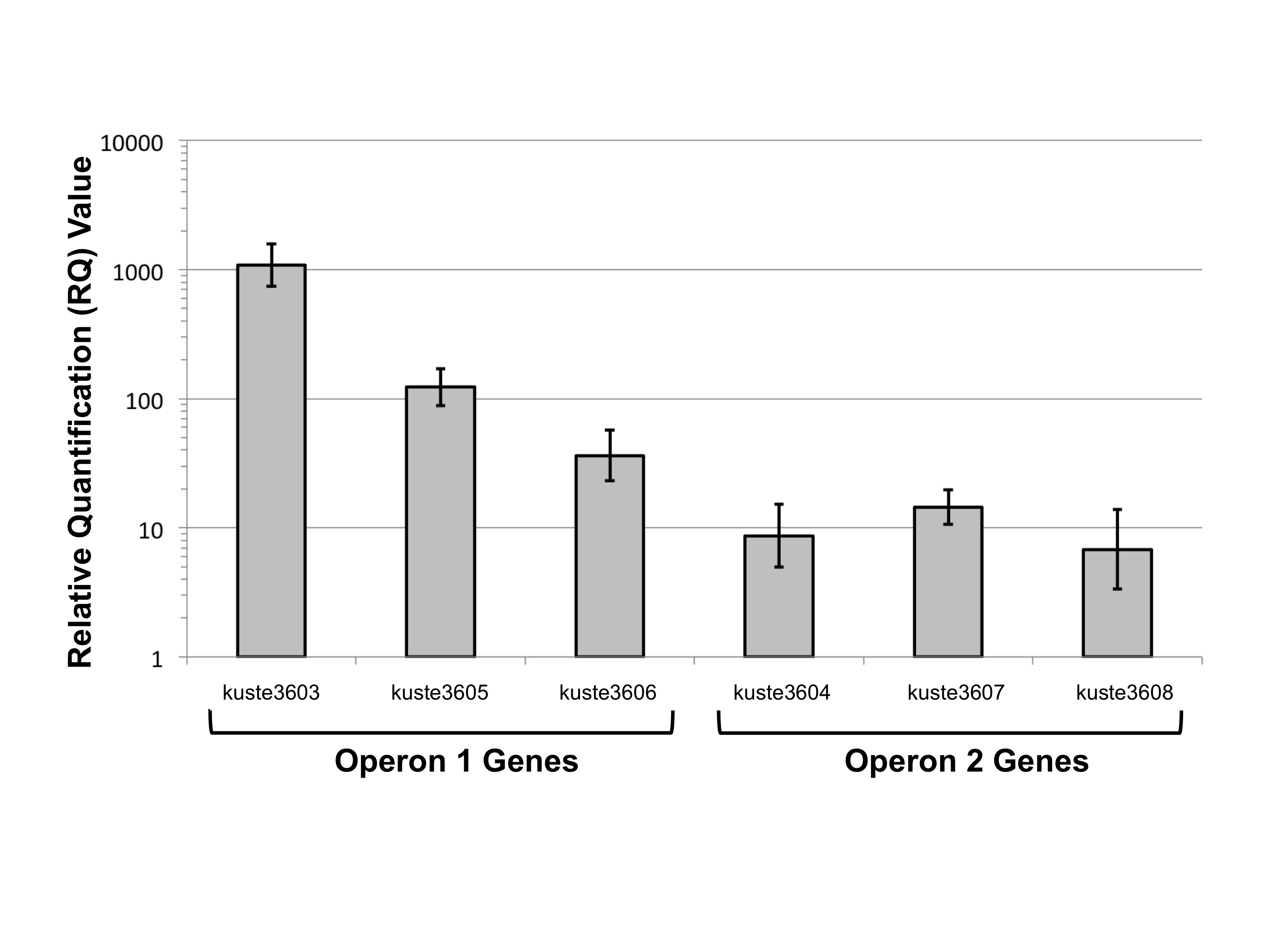

Supplement: S1 Fig — Fold-increase is given as relative quantification (RQ) value, obtained by the ΔΔCT method. Bars represent the range between the minimum and maximum RQ values. (TIF) [file pone.0151087.s001.tif]
