# Supplementary material for: Investigation of Proposed Ladderane Biosynthetic Genes from Anammox Bacteria by Heterologous Expression in E. coli
Source: PLoS One. 2016 Mar 14;11(3):e0151087. doi: 10.1371/journal.pone.0151087 (PMC4790861; doi:10.1371/journal.pone.0151087)
Supplement: S1 Table — (PDF) [file pone.0151087.s002.pdf]

S1 Table. Comparison of volumetric energy densities for fused cyclobutanes and petroleum-derived and renewable jet fuels

**Compound or fuel**	**Volumetric energy density (MJ/L)**
Cyclobutane, C_4H_8	33.0[a]
[2]-ladderane, C_6H_{10}	42.3[a]
[3]-ladderane, C_8H_{12}	46.3[a]
[4]-ladderane, $C_{10}H_{14}$	49.2[a]
[5]-ladderane, $C_{12}H_{16}$	51.4[a]
Jet fuel (kerosene)[b]	35.06[c]
Bio-SPK[b]	33.2[b]

[a] Calculated using entropies of formation (Novak 2008) and estimated densities from ACD/Labs' PhysChem Suite software

[b] This fuel is the most relevant to commercial aviation in the U.S. Bio-SPK, Bio-Derived Synthetic Paraffinic Kerosene, is a renewable jet fuel in use today (Kallio et al. 2014)

[c] From Hemighaus et al. (2004)

Hemighaus, G., et al. (2004) Aviation Fuels Technical Review, *Chevron Corporation*.

Kallio, P., et al. (2014) Renewable jet fuel, *Current Opinion in Biotechnology 26*, 50-55

Novak, I. (2008) Ring strain in [n]ladderanes, *J. Phys. Chem. A 112*, 10059-10063.
